# The Janus Head of Oxidative Stress in Metabolic Diseases and During Physical Exercise

**DOI:** 10.1007/s11892-017-0867-2

**Published:** 2017-04-24

**Authors:** Dominik Pesta, Michael Roden

**Affiliations:** 1 Institute for Clinical Diabetology, German Diabetes Center, Leibniz Center for Diabetes Research at Heinrich-Heine University Düsseldorf, Düsseldorf, Germany; 2 German Center for Diabetes Research (DZD e.V.), Munich, Neuherberg Germany; 3 Department of Endocrinology and Diabetology, Medical Faculty, Heinrich-Heine University, c/o Auf’m Hennekamp 65, 40225 Düsseldorf, Germany

**Keywords:** Reactive oxygen species, Antioxidant capacity, Obesity, Type 2 diabetes, Exercise

## Abstract

**Purpose of Review:**

Oxidative stress describes an imbalance between production and degradation of reactive oxygen species (ROS), which can damage macromolecules. However, ROS may also serve as signaling molecules activating cellular pathways involved in cell proliferation and adaptation. This review describes alterations in metabolic diseases including obesity, insulin resistance, and/or diabetes mellitus as well as responses to acute and chronic physical exercise.

**Recent Findings:**

Chronic upregulation of oxidative stress associates with the development of insulin resistance and type 2 diabetes (T2D). While single bouts of exercise can transiently induce oxidative stress, chronic exercise promotes favorable oxidative adaptations with improvements in muscle mitochondrial biogenesis and glucose uptake.

**Summary:**

Although impaired antioxidant defense fails to scavenge ROS in metabolic diseases, chronic exercising can restore this abnormality. The different metabolic effects are likely due to variability of reactive species and discrepancies in temporal (acute vs. chronic) and local (subcellular distribution) patterns of production.

## Introduction

Mitochondrial respiration generates reactive oxygen species (ROS), which are quenched by antioxidant systems. Various processes such as insulin signaling and upregulation of antioxidants, adaptive protein synthesis, and mitochondrial biogenesis depend on increased ROS generation under physiological conditions such as exercising. On the other hand, dysregulation of ROS production and removal, termed oxidative stress, occurs in numerous human disorders including type 2 diabetes (T2D) and obesity [1] and has been related to their pathogenesis and complications. This review will address detrimental effects of systemic ROS and ROS originating from skeletal muscle-inducing oxidative stress and cellular damage in the context of metabolic diseases but will also explore effects of different exercise training interventions on oxidative stress in this cohort. This review is based on a search in biomedical databases (PubMed, Quertle) for the terms “obese, insulin resistant, type 2 diabetes, ROS, human, oxidative stress” as well as “obese, insulin resistant, type 2 diabetes, acute, chronic, exercise, ROS, human, oxidative stress” and mainly focuses on studies published during the last 5 years but also addresses relevant older studies.

## What Is Oxidative Stress?

The term oxidative stress has first been introduced to the biomedical research community in 1985. Oxidative stress has originally been described as a disturbance in the pro-oxidant–antioxidant balance in favor of the former, potentially leading to cellular damage [2]. After the discovery of redox pathways, this definition has been rephrased as “a disruption of redox signaling and control” [3]. Oxidative stress can also be defined as a state of temporarily or chronically elevated ROS production, ROS production is temporarily or chronically elevated, perturbing cellular metabolism and damaging cellular components [4].

**Fig. 1:**
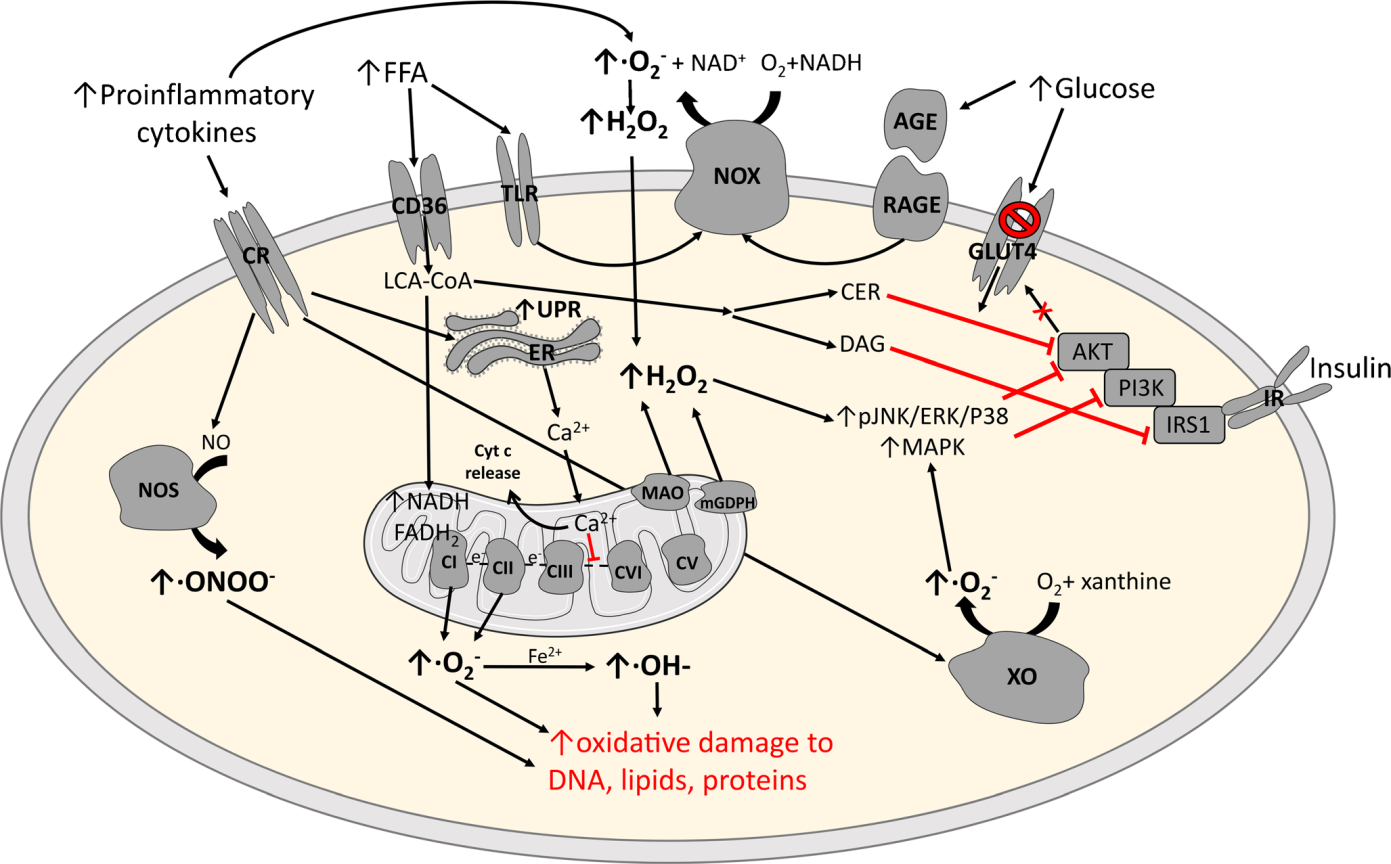
Pathological effects of chronic oxidative stress. Major sources of reactive oxygen species (ROS) involved in the pathophysiology of insulin resistance and obesity are shown. The factors responsible for elevated chronic oxidative stress include inflammatory processes via cytokine receptors (*CR*), increased free-fatty acids (FFA) via Toll-like receptor (TLR), and hyperglycemia, which promote ROS emission from the NADPH oxidase system (*Nox*) and nitric oxide (*NO*) synthase (*NOS*) as well as xanthine oxidase (*XO*) and the mitochondria. Binding of advanced glycation end products (*AGE*) to its receptor (*RAGE*) can further stimulate Nox-mediated ROS release. Increased mitochondrial oxidation from nutrient overload and participation of monoamine oxidase (*MAO*) and mitochondrial glyceraldehyde-3-phosphate dehydrogenase (*mGDPH*) can contribute to excess intracellular ROS production, which can cause oxidative damage to DNA, proteins, and lipids and also activate the mitogen-activated protein kinase (*MAPK*) pathway and C-Jun-N-terminal kinase (*JNK*), contributing to impaired insulin signaling and glucose uptake. Elevated intracellular diacylglycerols (*DAG*) and ceramides (*CER*) also interfere with insulin signaling. Endoplasmic reticulum (*ER*) stress via the unfolded protein response (*UPR*) remains an important source for increased ROS generation. Calcium release from the ER can enhance cytochrome c release and interfere with electron transfer within the electron transfer system, thereby further increasing mitochondrial ROS generation. *AKT* protein kinase B, *CI–CV* mitochondrial complex I–V, *ERK* extracellular signal-regulated kinase, *GLUT4* glucose transporter 4, *IR* insulin receptor, *IRS* insulin receptor substrate, *PI3K* phosphoinositide 3-kinase. *Arrows* denote activation, *red lines* indicate inhibition, and *words in red* represent the most important outcome of oxidative damage to cellular macromolecules

ROS are chemical species produced by sequential four-electron reduction of molecular O_2_ through the addition of electrons at metabolically active sites such as the mitochondria or cytosolic enzymes during their catalytic activity. They comprise of superoxide anion radical (O_2_·−), hydrogen peroxide (H_2_O_2_), and hydroxyl radical (OH^·−^), which are chemically instable and have a strong tendency to react with and damage biological molecules (Fig. 1). Caloric overload can stimulate substrate flux to the mitochondria, giving rise to electron donors (NADH, FADH_2_) and global electron transport activity with additional electron leakage due to high membrane potential (ΔΨ). Major sites of mitochondrial net ROS emission include complex I and complex III of the electron transfer system (ETS) in the inner mitochondrial membrane, the mitochondrial glyceraldehyde-3-phosphate dehydrogenase [5] in the mitochondrial matrix, as well as the flavoprotein monoamine oxidase in the outer mitochondrial membrane [6, 7]. Other ROS emission sites include the enzymes nicotinamide adenine dinucleotide phosphate (NADPH) oxidase (Nox) [8] and nitric oxide synthase (NOS) [9] as well as enzymes that produce ROS as a byproduct, such as xanthine oxidase (XO) and lipoxygenase [10]. The process of oxidative protein folding in the endoplasmic reticulum (ER) can also serve as an important source of ROS and contributes to approximately 25% of overall cellular ROS emission [11]. ER-mediated ROS emission due to oxidative protein folding increases during augmented demand in insulin-resistant individuals due to inflammation and higher insulin biosynthesis [12].

Another type of redox signaling molecule, reactive nitrogen species (RNS), is derived from ^·^NO, a byproduct of l-arginine-l-citrulline metabolism catalyzed by NOS isozymes. ^·^NO can react with superoxide to form the highly reactive peroxynitrite (ONOO^−^). Other RNS include the nitrogen dioxide radical (^·^NO_2_) and nitrite (NO_2_−). RNS may modulate cell signaling or damage cells by oxidation of biological macromolecules and nitrosylation of other proteins.

In order to detoxify these highly reactive molecules and to maintain low degrees of oxidative stress, the healthy cell relies on a large array of antioxidant defense mechanisms. They are responsible for scavenging and breaking down ROS to less or nonreactive products and include antioxidant enzymes such as superoxide dismutase (SOD), catalase (CAT), and glutathione peroxidase (GPx) as well as endogenous metabolites like bilirubin and uric acid [13]. There are three mammalian isoforms of SOD: cytosolic Cu/Zn SOD or SOD1, mitochondrial Mn-dependent SOD or SOD2, and extracellular Cu/Zn SOD or SOD3, which catalyze detoxification of O_2_^·−^ to oxygen and H_2_O_2_ [14]. CAT catalyzes the degradation of H_2_O_2_ to water and oxygen, while GPx reduces H_2_O_2_ and lipid hydroperoxides to water or corresponding alcohols consuming reduced glutathione (GSH) [15]. Obviously, both antioxidant defense and oxidant load affect the redox balance and insufficient scavenging may be an important cause for oxidative stress.

## Methods for Detecting Oxidative Stress

Previous reviews have comprehensively described the methods for assessment of oxidative stress [16–18]. Briefly, the challenge of measuring ROS results from their short half-life ranging from nanoseconds to seconds and their overall very low concentrations. ROS can be either detected directly or indirectly by measuring molecules that preferably react with ROS in vivo.

### Direct Methods to Assess Oxidative Stress

Highly specific methods involve the trapping of O_2_^·−^ with spin trapping reagents such as α-phenyl-*N*-*tert*-butyl nitrone (PBN) that covalently bind and form stable adducts with radicals and can be detected using quantitative electron spin resonance (ESR) [16]. Alternatively, rather unspecific spin probes can be used to detect O_2_^·−^ in intact tissues, cells, or homogenates. These probes are oxidized to form stable radicals, which are then detectable by ESR, a method often regarded as the gold standard, although methodological limitations, high costs, and extensive training impede its broad use [19].

Alternative approaches to detect ROS in cultured cells, tissues, or isolated mitochondria rely on colorimetic, fluorimetric, or luminescence-based assays as well as enzyme activity assays. These assays follow the principle that the radical reacts with a tracer, which creates a detector compound that releases a photon. The widely used quantitative lucigenin-enhanced chemiluminescence assay uses lucigenin, a compound reasonably specific for O_2_^·−^[19]. Although this assay is easy to use, it is prone to artifacts and the validity has been questioned based on O_2_^·−^ overestimation due to redox cycling of the compound [20]. Lucigenin at lower concentrations (5 μM) and other compounds such as coelenterazine, luminol, or methylated-modified cypridina luciferin analog that do not undergo redox cycling are promising probes for O_2_^·−^ detection [19].

The widely used Amplex Red assay measures extracellular H_2_O_2_ via the horseradish peroxidase-catalyzed reaction of *N*-acetyl-3,7-dihydroxyphenoxazine (Amplex Red) with H_2_O_2_ in a 1:1 stoichiometry to produce the red-fluorescent oxidation product, resorufin [21]. Although this assay is highly specific and sensitive, Amplex Red is light sensitive and thereby prone to artefactual formation of resorufin at high concentrations of 50 μM [22]. Lowering the concentrations to 10 μM and minimizing light exposure makes the Amplex Red assay an accurate, sensitive, and versatile way for detecting H_2_O_2_ emission from cells, tissues, and cell-free systems.

### Indirect Methods to Assess Oxidative Stress

The reaction of thiobarbituric acid (TBA) with the end product of lipid peroxidation, malondialdehyde (MDA), is among the earliest and most widely used methods for quantitative detection of lipid peroxides [23]. Although the assay is fast and technically easy to perform, TBA can also react with other saturated and unsaturated aldehydes to form unspecific TBA-reactive substances (TBARS) and possibly overestimate MDA levels. Separation of the aldehyde adducts by high-performance liquid chromatography (HPLC) has therefore been applied to improve the sensitivity and accurately quantify MDA levels in tissues and plasma [24]. Isoprostanes are other important markers of lipid peroxidation, which can be detected in all body fluids including urine [25]. F_2_-isoprostane, the product of peroxidation of arachidonic acid, is considered to be very accurate to quantify in vivo oxidative stress in plasma or urine [26].

8-Hydroxy-2-deoxyguanosine (8-OHdG), the main product of DNA oxidation, can easily be assessed in human DNA samples and in urine by HPLC, gas chromatography mass spectrometry (GC/MS), or enzyme-linked immunosorbent assay (ELISA) [27]. Difficulties arise from the formation of artifacts during isolation and analysis of DNA and from confounding factors such as smoking [28]. Nevertheless, 8-OHdG is an important marker for measuring the effect of endogenous oxidative damage to DNA as a biomarker of oxidative stress.

ROS-mediated protein oxidation leads to formation of carbonyl groups (aldehydes and ketones) on protein side chains, mainly of proline, arginine, lysine, and threonine [29]. These stable carbonylated proteins can be detected after derivatization of the carbonyl group with 2,4-dinitrophenylhydrazine (DNPH) and formation of dinitrophenyl (DNP) hydrazone [29]. Hydrazones are measured spectrophotometrically by ELISA and Western blotting, the latter yielding semiquantitative results. Appropriate sample handling and performing the measurement as quickly as possible help to minimize artifact formation during sample collection and analysis. Although carbonylated proteins are induced by various chemical processes and different ROS, their relatively early formation and stability (hours to days) as compared to lipid peroxidation products (minutes) make them a valuable biomarker for oxidative stress [30].

The wide array of methods to detect ROS in different biological matrices offers not only a variety of advantages but also potential drawbacks. Of note, none of these methods is generally suitable for every condition. It is therefore recommended to use at least two independent methods to improve the consistency of experimental observations regarding oxidative stress. The choice should be made considering the sensitivity of the assay in the tested biological specimen. Interest in more general effects of oxidative stress will lead to the use of less specific fluorescent probes such as Amplex Red or indirect methods to detect oxidized macromolecules. Interest in the effects of certain radical species will call for ERS or lucigenin assays. New methods such as genetically encoded ROS reporters, nanoparticle delivery systems, and nanotube ROS probes will likely advance the field by enhanced specificity and sensitivity as well as localization of radical generation [31].

## Links Between Oxidative Stress and Metabolic Diseases

John Baynes was one of the first to present the hypothesis that oxidative stress could be an important mechanism contributing to the pathogenesis of diabetes, arguing that diabetes-related complications are associated with oxidative damage of proteins and lipids [32]. Subsequent studies supported this hypothesis in that increased systemic and skeletal muscle ROS production may relate to the development of several metabolic abnormalities including obesity and T2D [33, 34].

### Systemic Oxidative Stress in Obesity and Insulin Resistance

Several studies reported mainly elevated oxidative stress in metabolic disorders in humans (Table 1). In obese nondiabetic humans, different parameters of fat accumulation correlate with systemic oxidative stress [35, 36]. Similarly, oxidative DNA damage is elevated in individuals with prediabetes, defined by impaired fasting blood glucose >6 mmol/l but <7 mmol/l [37]. While antioxidant capacity (SOD, CAT) is also upregulated in young obese individuals, elderly obese persons and individuals with metabolic syndrome show decreased antioxidant capacity in the face of increased systemic lipid peroxidation and protein carbonyls in parallel with altered lipoprotein metabolism and decreased antioxidant capacity, and both oxidative stress and antioxidant capacity (SOD, CAT) are upregulated in young obese individuals [38, 39]. Thus, systemic oxidative stress is present in obese and insulin-resistant individuals, which further rises with aging and progression of metabolic abnormalities due to inadequate upregulation of antioxidant defense.

**Table 1:** Effects of ROS on the systemic and cellular environment in human obesity, insulin resistance, and/or type 2 diabetes (studies are listed in chronological order)

Author	Population	Biological matrix	Pro-oxidants	Antioxidants
Shin et al. [49]	T2D (*n* = 41) HC (*n* = 33)	Serum	↑8-OHdG	n.a.
Kanauchi et al. [48]	T2D (*n* = 25) HC (*n* = 20)	Urine	↑8-OHdG	n.a.
Bruce et al. [54]	T2D (*n* = 7) oHC (*n* = 5) yHC (*n* = 9)	Vastus lateralis muscle	n.a.	↓HSP72; ↓HO-1 T2D ↔HSP72 and HO-1 vs. yHC
Furukawa et al. [35]	HC (*n* = 140)	Plasma and urine	↑TBARS, F_2_-isoprostane associated with body fatness	n.a.
Dave and Kalia [52]	T2D (*n* = 50) HC (*n* = 50)	Erythrocytes and plasma	↑TBARS	↓GPx, CAT, GSH
Silver et al. [40]	OB/OW (*n* = 42) HC (*n* = 39)	Endothelial cells	↑Nox	↑CAT, SOD
Song et al. [51]	T2D (*n* = 113) IGR (*n* = 78) HC (*n* = 92)	Erythrocytes and plasma	↑MDA in T2D ↑DNA damage in T2D and IGR	↓SOD in IGR vs. HC ↓SOD and TAC in T2D vs. HC
Anderson et al. [42]	Male OB-IR (*n* = 3) HC (*n* = 5)	Vastus lateralis muscle	↑H_2_O_2_ emission	↓GSH/GSSG
Abdul-Ghani et al. [43]	T2D (*n* = 10) OB-IR without T2D (*n* = 10) HC (*n* = 10)	Vastus lateralis muscle	↓H_2_O_2_ emission in OB-IR ↔in T2D and HC ↑H_2_O_2_/ATP ratio in T2D	n.a.
Park et al. [47]	HC (*n* = 5115)	Plasma	OxLDL and F_2_-isoprostanes associated with IR	n.a.
Lefort et al. [41]	OB-IR (*n* = 14) HC (*n* = 20)	Vastus lateralis muscle	↑H_2_O_2_ emission ↔mito respiration ↓complex I subunits	n.a.
Al-Aubaidy and Jelinek [37]	T2D (*n* = 35) PRE (*n* = 8) HC (*n* = 119)	Serum	↑8-OHdG in T2D and PRE vs. HC	n.a.
Karaouzene et al. [39]	yOB (*n* = 45) oOB (*n* = 40) yHC (*n* = 65) oHC (*n* = 55)	Erythrocytes and plasma	↑hydro peroxides in OB ↑protein carbonyls in OB	↓Total antioxidant capacity in OB ↑SOD, CAT in yOB ↓SOD, CAT in oOB ↓GPx in OB
Bravard et al. [53]	T2D (*n* = 10) OB (*n* = 10) HC (*n* = 10)	Vastus lateralis muscle	↑protein carbonyls in T2D ↑ROS in FTO overexpressing cells	↓SOD2 in T2D
Codoner-Franch et al. [44]	yOB (*n* = 40)	Plasma	Lipid peroxidation and protein carbonyls correlate with IR	n.a.
Yokota et al. [38]	MS (*n* = 14) HC (*n* = 13)	Plasma and serum	↑TBARS	↓total thiols ↓SOD
Warolin et al. [36]	African American (*n* = 82) White American (*n* = 76)	Urine	F_2_-isoprostane positively correlated with body fatness ↔between groups	n.a.
Ohara et al. [55]	T2D (*n* = 68)	Plasma	↑d-ROMs associated with daily glucose variability	n.a.
Kant et al. [50]	T2D or prediabetes (*n* = 43) HC (*n* = 37)	Urine	↑8-OHdG, S-cdA, and 8-iso-PGF_2α_	n.a.

*8-OHdG* 8-hydroxy-2′-deoxyguanosine, *8-iso-PGF*_*2α*_ 8-iso-prostaglandin F_2α_, *ATP* adenosine triphosphate, *CAT* catalase, *CS* citrate synthase, *DNA* deoxyribonucleic acid, *d-ROMs* diacron-reactive oxygen metabolites, *FTO* fat mass and obesity associated, *GPx* glutathione peroxidase, *GSH* glutathione, *GSSG* glutathione disulfide, *HC* healthy controls, *HSP* heat shock protein, *IGR* impaired glucose regulation, *IR* insulin resistant, *MDA* malondialdehyde, *MS* metabolic syndrome, *n.a.* not assessed, *Nox* nitric oxide synthase, *o* old, *OB* obese individuals, *OW* overweight individuals, *ROS* reactive oxygen species, *SOD* superoxide dismutase, *S-cdA* (5′S)-8,5′-cyclo-2′-deoxyadenosines, *T2D* individuals with type 2 diabetes, *TBARS* thiobarbituric acid reactive substances, *y* young

### Tissue-Specific Oxidative Stress in Obesity and Insulin Resistance

In a healthy cohort covering a wide range of body masses (body mass index (BMI) 18 to 37 kg/m^2^), vascular oxidative stress, and expression of Nox-p47^phox^, a Nox accessory protein involved in O_2_^·−^ production rises with the degree of adiposity without alteration of xanthine oxidase activity [40] (Table 1). The overweight/obese group features elevated protein carbonyls despite higher vascular CAT and SOD expression, suggesting compensation for increased oxidative stress. Notably, systemic plasma markers of oxidative stress and antioxidants are not different between normal weight and overweight/obese individuals in this study [40]. Obese insulin-resistant individuals exhibit twofold higher muscle mitochondrial H_2_O_2_ emission than healthy controls, possibly related to lower protein abundance of complex I subunits as well as enzymes responsible for the oxidation of fatty acids and branched-chain amino acids [41]. A similar increase in muscle mitochondrial H_2_O_2_ emission is paralleled by a 50% reduction in the GSH/GSSG ratio in muscles of obese insulin-resistant individuals [42]. Of note, others report unchanged or even decreased rates of H_2_O_2_ emission in obese insulin-resistant individuals, but these results are difficult to interpret in the absence of data on maximal ROS production rates [43]. Nevertheless, the above observations suggest that increased fat mass and insulin resistance may favor ROS production from vascular endothelium and skeletal muscle with insufficient tissue-specific compensation by antioxidant systems, leading to increased systemic oxidative stress independent of age and hyperglycemia.

### Associations of Fat Mass and Insulin Resistance With Oxidative Stress

Obese children with greater insulin resistance as assessed by the homeostasis model assessment of insulin resistance (HOMA-IR) also present with a higher degree of oxidative stress [44]. In addition to insulin resistance, circulating triglycerides and high-sensitivity C-reactive protein (hsCRP) may be involved in the relationship between adiposity and oxidative stress as adjustment for these parameters reduces the association. Indeed, elevated hsCRP strongly associates with oxidative stress independent of BMI and insulin resistance [45, 46]. Furthermore, plasma adiponectin levels are negatively associated not only with BMI and waist circumference but also with markers of systemic oxidative stress. In a population-based observational study of 5115 individuals, the positive relationship of levels of oxidative stress markers with HOMA-IR disappears for F_2_-isoprostane after adjustment for adiposity but remains for oxidized low-density lipoprotein (ox-LDL) [47].

Taken together, these results suggest that fat accumulation, insulin resistance, and deranged lipoprotein metabolism can contribute to increased oxidative stress independent of hyperglycemia. However, these studies do not allow to draw conclusions on causal relationships.

### Systemic Oxidative Stress in T2D

T2D patients show elevated markers of oxidative DNA damage in plasma and urine [48, 49] (Table 1). Of note, oxidative DNA damage and lipid peroxidation are already present in newly-diagnosed T2D and even in prediabetic individuals, defined as fasting blood glucose between 5.5 and 7 mmol/l and hemoglobin HA1c (HbA1c) levels of 5.7 to 6.4% [50]. While individuals with impaired glucose regulation (impaired fasting glucose and impaired glucose tolerance) show similar levels of oxidative stress, but slightly reduced erythrocyte SOD activity compared to glucose-tolerant people, levels of plasma lipid peroxides, and DNA damage are elevated and total antioxidant capacity, GPx, GSH, and SOD activity are decreased in T2D [51, 52].

### Tissue-Specific Oxidative Stress in T2D

Oxidative tissue damage measured as increased protein carbonyls is present in the skeletal muscle of T2D individuals in parallel with decreased SOD activity [53] (Table 1). Also, expression of heat shock protein (HSP)72 and heme oxygenase (HO)-1, genes responsible for antioxidant defense mechanisms, is markedly lower in the skeletal muscle of T2D [54] (Table 1). In T2D, muscle expression of antioxidant genes further correlates with muscle oxidative capacity and insulin-stimulated glucose disposal [54, 55].

Collectively, these results suggest that even moderate increases in blood glucose impair antioxidant defense, which leads to oxidative damage with possible deterioration of skeletal muscle function in overt diabetes.

### Associations of Hyperglycemia and Diabetes With Oxidative Stress

The reduction in antioxidant defense associates negatively with whole-body insulin sensitivity. Markers of oxidative DNA damage correlate with BMI, hyperglycemia, and β-cell dysfunction and progressively increase from prediabetic (5.5 and 7 mmol/l) to diabetic conditions [37, 51]. On the other hand, the positive correlation of ox-LDL and lipid peroxidation and the negative correlation of total antioxidant levels and SOD activity with insulin resistance were found to be independent of obesity in one study [47]. In T2D, muscle expression of antioxidant genes further correlates with muscle oxidative capacity and insulin-stimulated glucose disposal [54, 55]. Finally, oxidative stress is further associated with daily and day-to-day glucose variability [54, 55]. Taken together, glycemic control is an important driving force for further accelerating oxidative stress and impairment of antioxidant defense.

### Results From Diet Intervention Studies

Ingestion of a high-fat diet increases mitochondrial H_2_O_2_ emission and induces insulin resistance in healthy males [42], but not in obese insulin-resistant women [56•]. High-fat diet-induced oxidative stress in skeletal muscle also associates with reduced expression of muscle mitochondrial oxidative phosphorylation genes [42, 57]. However, comparison of a high-carbohydrate and a high-fat meal reveals that only the high-carbohydrate meal decreases total antioxidant capacity and muscle SOD, supporting the view of the deleterious role of carbohydrates and glycemia for oxidative stress [58].

On the other hand, diet-induced weight loss can decrease oxidative stress by improving antioxidant status independent of physical activity [59]. In obese women, body weight reduction not only improves insulin resistance, oxidative stress, and inflammation but also activities of antioxidant enzymes including GSH and CAT [60, 61]. Similarly, a 2-month calorie restriction by 20% resulting in 8% weight loss leads to reduction in dyslipidemia as well as markers of oxidative stress and inflammation along with improved antioxidant defense [62]. Taken together, intervention studies suggest that caloric intake and body weight changes dynamically affect oxidative stress but do not allow to identify whether oxidative stress contributes to the weight-dependent alterations in metabolism and insulin resistance.

### Cellular Mechanisms Contributing to ROS Production in Human Metabolic Diseases

At least 0.2–2% of the oxygen consumed during mitochondrial respiration contributes to the generation of free radicals [63, 64] (Fig. 1). T2D patients exhibit slightly lower flux through muscle ATP synthesis as well as in muscle expression of genes involved in mitochondrial function and oxidative metabolism [65–67]. Incomplete mitochondrial catabolism of long-chain fatty acyl-CoA has been further associated with elevated ROS production and impaired glutathione antioxidant system [68]. Intracellular accumulation of lipid metabolism intermediates (diacylglycerols, ceramides) further impairs insulin signaling [69••]. However, the data on muscle mitochondrial function in insulin-resistant (IR) and T2D humans are not fully consistent in that some features of mitochondrial function are comparable between T2D and age-matched glucose-tolerant individuals when respiratory rates are normalized to mitochondrial DNA or citrate synthase activity.

Adipose tissue may serve as an important source of ROS: nondiabetic obese KKay mice exhibit increased lipid peroxidation and H_2_O_2_ production in white adipose tissue. Elevated circulating fatty acids could contribute to oxidative stress via NADPH oxidase activation in white adipose tissue [35]. Obesity also relates closely to ER stress [70], which, in turn, associates with oxidative stress [71]. ER-mediated ROS production is increased in both obese insulin-resistant nondiabetic persons and T2D patients [72, 73]. In the context of ER stress, unfolded protein response (UPR) leads to calcium ion leakage from ER, which interferes with electron transfer in the ETS [74] and the subsequent cytochrome c release from mitochondria can induce mitochondrial ROS production [75] (Fig. 1).

Finally, advanced glycation end products (AGEs) increase under conditions of hyperglycemia [76]. Binding of AGEs to their respective receptors (RAGE) stimulates Nox, which also generates intracellular ROS [77]. (Fig. 1).

## Effects of Acute and Chronic Exercise on Oxidative Stress in Metabolic Diseases

Since the 1970s, it is known that 1 h of moderate endurance exercise can increase lipid peroxidation in humans [78]. Although the biological meaning was unknown, these results created a lot of interest over the following years about the role ROS plays during exercise. While acute exercise may induce a temporary state of oxidative stress, chronic physical activity promotes favorable oxidative adaptations [79]. Various modalities of regular exercising (endurance, resistance, or combined training) generally improve systemic markers of oxidative stress and antioxidant capacity in healthy individuals [80, 81], but its impact on oxidative stress in metabolic diseases is less clear.

### Acute Exercise and Oxidative Stress in Metabolic Diseases

An acute bout of exhaustive aerobic exercise results in greater ROS production in obese than in nonobese individuals [82] (Table 2). Both intensive aerobic and resistance exercise sessions lead to excessive lipid peroxidation in obese men and women [83, 84]. However, low-intensity exercise such as walking decreases lipid peroxidation in individuals with T2D, suggesting that mild exercising is able to reduce systemic oxidative stress in T2D [85]. Of note, acute high-intensity exercise-induced oxidative stress associates with increased insulin sensitivity in obese individuals [86]. Experimental studies in myocytes mimicking acute and chronic oxidative stress support this concept [87]. During acute and oxidative stress, the mitogen-activated protein kinase (MAPK) phosphatase MKP7 relocates from the nucleus to the cytoplasm, where it dephosphorylates JNK in the cytoplasm, resulting in increased insulin sensitivity through insulin receptor substrate (IRS)-1. These results suggest that oxidative stress in response to exercise is exacerbated in individuals with metabolic diseases but may also serve as an important signal improving insulin signaling and mitochondrial biogenesis.

**Table 2:** Effects of acute and chronic exercise interventions on ROS and energy metabolism in humans with metabolic diseases (studies are listed in chronological order)

Author	Population	Intervention (duration; mode; frequency)	Biological matrix	Pro-oxidants	Antioxidants
Acute exercise					
Vincent et al. [83]	OB (*n* = 14) HC (*n* = 14)	AE, RE	Plasma	↑lipid peroxides in OB after AE and RE	↑antioxidant capacity in HC after RE
Vincent et al. [84]	OW (*n* = 24) HC (*n* = 8)	AE	Plasma	↑lipid peroxides in OW after AE	↔thiols
Roh et al. [82]	OB (*n* = 12) HC (*n* = 12)	AE	Plasma	↑ROS in OB	↑SOD after AE
Haxhi et al. [85]	T2D (*n* = 9)	AE	Urine	↓F_2_-isprostanes	n.a.
Parker et al. [86]	OB (*n* = 11)	HIIT	Vastus lateralis muscle and plasma	↑JNK/MAPK	↑insulin-stimulated SOD
Chronic exercise					
Kasimay et al. [99]	OB-IGT (*n* = 14)	12 weeks; CR + AE; 3/week	Plasma	↓lipid peroxides	↑SG
Gutierrez-Lopez et al. [89]	OB (*n* = 32) HC (*n* = 16)	12 weeks; CR, CR + AE; 3/week	Plasma	↓lipid peroxides, protein carbonyls	n.a.
Brinkmann et al. [101]	T2D (*n* = 15) OB (*n* = 12)	12 weeks; AE; 3/week AX before and after	Plasma, erythrocytes	↓F_2_-isprostanes with AE, ↔with AX in T2D and OB	↑peroxiredoxin oxidation in T2D with AX after AE
de Oliveira et al. [97]	T2D (*n* = 43)	12 weeks; AE, RE, CT, NT; 3/week	Plasma	↔lipid peroxides	↑CAT, SOD, NO, SG with AE; ↔with RE; ↑SG with CT
McNeilly et al. [98]	OB-IGT (*n* = 11)	12 weeks; mild AE; 5/week	Serum	↓lipid peroxides	↔SOD
Krause et al. [102]	T2D (*n* = 13) HC (*n* = 12)	16 weeks; AEL, AEM; 3/week	Plasma and vastus lateralis muscle	↑protein carbonyls in T2D; ↑NO, NOS in HC	↑CAT in T2D with AEM
Vinetti et al. [96••]	T2D (*n* = 20)	52 weeks; RE, AE, FL; 3/week	Plasma and PBMC	↓lipid peroxides	n.a.
Pittaluga et al. [94]	T2D (*n* = 12) HC (*n* = 12)	12 weeks; AE, 3/week	Plasma	↓MDA, DNA damage in T2D	↑GSH, AA
Medeiros et al. [92]	OB (*n* = 25)	5 weeks; CT1; 5/week 9 weeks; CT2; 3/week	Plasma	↓protein carbonyls CT1 ↑protein carbonyls CT2	↓GPx
Gram et al. [93•]	yHC (*n* = 17) eHC (*n* = 15)	2 weeks IM followed by 6 weeks; AE; 3/week	Vastus lateralis muscle	↑H_2_O_2_ emission, ↓ATP generation after IM, reversed by AE	↑CAT, SOD with AE
Bianchi et al. [88]	OOW (*n* = 50)	12 weeks; CR + AE; 3/week	Plasma	↓hydroperoxides	n.a.
Farinha et al. [91]	MSW (*n* = 23)	12 weeks; AE; 3/week	Plasma and serum	↓lipid peroxides ↓protein carbonyls	↑total thiols
Konopka et al. [56•]	OW (*n* = 25) HC (*n* = 14)	12 weeks; AE; 3/week	Vastus lateralis muscle	↓H_2_O_2_ emission and DNA damage	↑CAT
Dincer et al. [95]	T2D (*n* = 31)	12 weeks; AE; 3/week	Plasma	↓protein carbonyls	↑sialic acid
Duggan et al. [90]	OOW (*n* = 439)	52 weeks; CR, AE, CR + AE, NT; 3/week	Plasma	↓F_2_-isprostanes in CR and CR + AE, ↔in AE	n.a.
Karstoft et al. [103]	T2D (*n* = 14)	2 weeks; IW, CW, NT; 5/week	Plasma, urine	↔F_2_-isprostanes	n.a.
Dantas et al. [81]	HEW (*n* = 25)	10 weeks; RE; 3/week	Plasma	↓MDA	↑H_2_O_2_ scavenging

*AA* ascorbic acid, *AE* aerobic exercise, *AEL* aerobic exercise low, *AEM* aerobic exercise moderate, *AX* acute exercise, *CAT* catalase, *CR* calorie restriction, *CT* concurrent training, *CW* continuous walking, *GPx* glutathione peroxidase, *GSH* glutathione, *HC* healthy controls, *HEW* hypertensive elderly women, *IGR* impaired glucose regulation, *IGT* impaired glucose tolerance, *IM* immobilization, *IW* intermittent walking, *MDA* malondialdehyde, *MSW* women with metabolic syndrome, *n.a.* not assessed, *NO* nitric oxide, *NOS* nitric oxide synthase, *NT* no treatment, *OB* obese, *OOW* overweight/obese women, *PBMC* peripheral blood mononuclear cells, *RE* resistance exercise, *SG* sulfhydryl groups, *SOD* superoxide dismutase

### Chronic Exercising and Systemic Oxidative Stress in Obesity and Insulin Resistance

In overweight-to-obese women, 12 weeks of aerobic exercise training plus caloric restriction lowers systemic lipid peroxidation [88] (Table 2). In a similar 12-week intervention study, combined aerobic exercise and hypocaloric diet is more effective to decrease oxidative stress and improve serum antioxidant capacity than hypocaloric diet alone [89]. On the other hand, obese women feature reduction in markers of lipid peroxidation upon aerobic exercising plus caloric restriction or caloric restriction alone, but only in those individuals increasing their maximal oxygen uptake upon exercising alone [90]. Both regular aerobic and resistance training alone can improve oxidative stress and antioxidant defense in overweight individuals [81, 91]. Five weeks of combined resistance and aerobic training improve both oxidative stress and insulin resistance in insulin-resistant humans [92]. However, the combined exercise training can lead to higher oxidative lipid damage in obese individuals [92], suggesting that caution may be required when recommending combined training in persons with metabolic diseases.

### Chronic Exercising and Tissue-Specific Oxidative Stress in Obesity and Insulin Resistance

As little as 2 weeks of immobilization diminishes muscle ATP production and increases muscle H_2_O_2_ emission without effects on antioxidant proteins, while a subsequent 6-week period of aerobic training not only restores ATP production and H_2_O_2_ emission to baseline levels but also increases SOD and CAT [93•] (Table 2). Likewise, a 12-week aerobic exercise training period reverses muscle mitochondrial alterations, diminishes cellular oxidative damage, and increases CAT activity in obese insulin-resistant women despite minimal weight loss [56•].

### Chronic Exercising and Systemic Oxidative Stress in T2D

Chronic aerobic exercise training can reduce oxidative damage to proteins, lipids, and DNA as well as improve systemic antioxidant status in obese and T2D individuals [90, 94, 95] (Table 2). A 12-month supervised exercise training period consisting of aerobic, resistance, and flexibility training reduces some features of oxidative stress independent of changes in body weight [96••], but not systemic lipid peroxidation [97]. In obese individuals with impaired glucose tolerance, 12-weeks of mild aerobic training decreases body mass, percent body fat, and systemic lipid peroxidation and improves insulin sensitivity without affecting SOD [98]. As in insulin-resistant persons, aerobic training in combination with caloric restriction reduces oxidative stress and improves antioxidant capacity in obese glucose-intolerant persons [99]. The precise interaction between these two interventions remains to be established, as exercise training seems to provide no additional benefit when used in combination with caloric restriction [100]. Although individuals with T2D show elevated oxidative stress after maximal-intensity exercise compared to healthy individuals, 12 weeks of preconditioning with regular aerobic training markedly diminished oxidative stress in response to an acute bout of exercise in individuals with T2D [101].

### Chronic Exercising and Tissue-Specific Oxidative Stress in T2D

Only a few studies have addressed the effect of exercise on muscle oxidative stress in T2D (Table 2). A 16-week period of unsupervised aerobic exercise training at moderate intensity is more effective to attenuate muscle oxidative protein damage and to increase CAT activity in obese T2D patients than exercise training at low intensity [102]. These effects occurred without changes in insulin sensitivity and body composition. On the other hand, reductions in oxidative stress related to improvements in insulin sensitivity [96••]. In another study, only interval walking training but not continuous walking training improved glycemic control without any effect on oxidative stress in T2D patients [103]. These findings indicate that the training response of oxidative stress and antioxidant defense is dependent on intensity, at least at lower levels of exercising, but again does not necessarily relate to insulin sensitivity or glycemic control.

Although the effect of exercise on oxidative stress is somewhat mixed and dependent on the metabolic disease, individuals with metabolic diseases may experience an exacerbation of oxidative stress following exercise when compared to healthy individuals. However, this increased oxidative stress may act as a preconditioning and induce upregulation in antioxidant defenses, which leads to diminished levels of oxidative stress when experiencing subsequent pro-oxidant environments [104].

### Cellular Mechanisms Contributing to Exercise-Mediated ROS Production in Human Metabolic Diseases

In contrast to chronic oxidative stress in metabolic diseases, exercise-induced ROS occurs transiently, mostly limited to the duration of the exercise session. Thus, the pattern of ROS production follows the concept of hormesis, i.e., favorable biological effects at low exposures and opposite effects at higher doses. Adaption in response to exercise-induced oxidative stress renders the cell less vulnerable to successive perturbations [105]. ROS produced during higher-intensity exercise possibly promotes glucose uptake and improves glycemic control. In the mouse, repetitive contractions of extensor digitorum longus muscle stimulate glucose uptake by 300%. Contraction-stimulated, but not basal glucose uptake decreases by 50% after the addition of the antioxidant *N*-acetyl cysteine (NAC) [106]. Treatment with ebselen, an antioxidant reducing H_2_O_2_, similarly reduces glucose uptake, indicating a role of H_2_O_2_ generation [107]. Interestingly, these results could not be confirmed during in situ studies of NAC infusion in rats [108] and humans [109], possibly due to different NAC concentrations. Alternatively, ROS may only influence contraction-mediated glucose uptake during higher-intensity exercises with greatest ROS production [110]. Of note, treatment with the ROS scavengers and vitamins C and E surprisingly prevents exercise-induced increases in insulin sensitivity in healthy individuals, suggesting a beneficial role of ROS under exercise conditions [111].

Exercise-induced ROS also stimulates mitochondrial biogenesis and can improve mitochondrial function. Peroxisome proliferator-activated receptor gamma coactivator 1 alpha (PGC-1α) is a key regulatory factor mediating mitochondrial biogenesis [112], a process which also involves activation of AMP-activated protein kinase (AMPK) [113] (Fig. 1). Indeed, H_2_O_2_ treatment of C2C12 cells results in enhanced AMPK activation and PGC-1α promotor activity, both of which are blocked by NAC [114].

In summary, ROS generated during exercise likely mediates favorable adaptations including improvements in glucose uptake and mitochondrial biogenesis.

## Conclusions

Detecting oxidative stress remains challenging, although novel developments may improve the diagnostic efficacy of ROS measurement. Nevertheless, reliable and easy-to-use biomarkers of redox homeostasis will be required to assess oxidative stress in clinical studies.

Numerous studies support the concept of a compromised balance between ROS generation and the antioxidant defense network in obesity and insulin-resistant states. The resulting chronic oxidative stress contributes to insulin resistance. Exercise training can ameliorate these effects and result in adaptive responses and improved endogenous antioxidant capacity in individuals with metabolic diseases. These changes can occur independent of relevant weight loss. Although acute exercise might induce a short-term pro-oxidative environment, regular exercise, regardless of the modality, improves cellular antioxidant capacity in obese, T2D, and IR individuals. Chronic exercise training can reduce oxidative stress, but this reduction is not necessarily related to improved insulin sensitivity and/or glycemic control.

However, the precise localization and origin of ROS in different pathological and physiological conditions and the effect of specific ROS on specific signaling pathways remain unclear. Specifically, comprehensive human studies exploring triggers for ROS generation during acute and chronic exercise and the impact of ROS on important cellular signaling pathways in the context of exercise adaptation and development of T2D are still lacking.
